# Novel sheath device for pancreatic biopsies in intraductal papillary mucinous carcinoma with parenchymal invasion

**DOI:** 10.1055/a-2178-4143

**Published:** 2023-10-24

**Authors:** Fumitaka Niiya, Masataka Yamawaki, Jun Noda, Tetsushi Azami, Yuichi Takano, Fumiya Nishimoto, Masatsugu Nagahama

**Affiliations:** Division of Gastroenterology, Department of Internal Medicine, Showa University Fujigaoka Hospital, Japan

Preoperative evaluation of intraductal papillary mucinous carcinoma is challenging because “high-risk stigmata” in international guidelines are not always malignant 1 . Surgery is strongly recommended if pancreatic parenchymal invasion can be confirmed histologically 2 . Transpapillary biopsy for intraductal papillary mucinous neoplasms improves diagnostic yields 3 ; nonetheless, it is difficult to diagnose parenchymal invasion on small biopsy specimens and the pancreatitis risk due to the repeated insertion of biopsy forceps into the main pancreatic duct cannot be ignored.

The novel sheath device (Endosheather; Piolax, Kanagawa, Japan) consists of a tapered inner sheath, and an outer sheath ( Fig. 1 ). Biopsy forceps can be advanced through the outer sheath, and specimens can be repeatedly obtained without papillary injury from repeated forceps insertions.

**Fig. 1:**
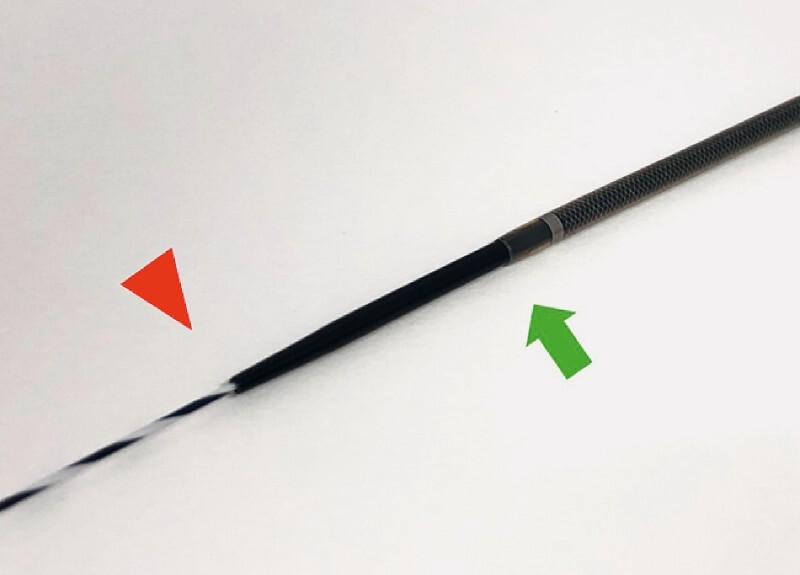
This device has an inner catheter with a tapered tip (red arrowhead) and an outer sheath (green arrow).

A 78-year-old woman was referred for main pancreatic duct dilation detected using computed tomography ( Fig. 2 a ). Endoscopic ultrasonography revealed a hypoechoic papillary tumor (16 mm) in the main pancreatic duct of the pancreatic head ( Fig. 2 b ). Endoscopic retrograde cholangiopancreatography was performed to biopsy the tumor. Following the insertion of a guidewire into the main pancreatic duct, an intraductal pancreatic tumor was detected through pancreatography ( Fig. 3 a ). A novel sheath device was inserted over the guidewire, and forceps (Radial Jaw 4 Pediatric Biopsy Forceps; Boston Scientific, Marlborough, Massachusetts, USA) were inserted into the main pancreatic duct through the sheath device to obtain the tumor specimen ( Fig. 3 b ). Finally, we obtained substantial tissue samples by performing three biopsies and placed a 6-Fr endoscopic nasopancreatic drainage tube ( Video 1 ). No adverse events were reported. Histologically, the specimen was diagnosed as intraductal papillary mucinous carcinoma with invasion of the pancreatic parenchyma ( Fig. 4 ), and a pancreaticoduodenectomy was scheduled. This novel sheath device is useful for obtaining substantial intraductal tissue samples without causing papillary edema due to the frequent insertion of biopsy forceps ( Video 1 ).

**Fig. 2 a:**
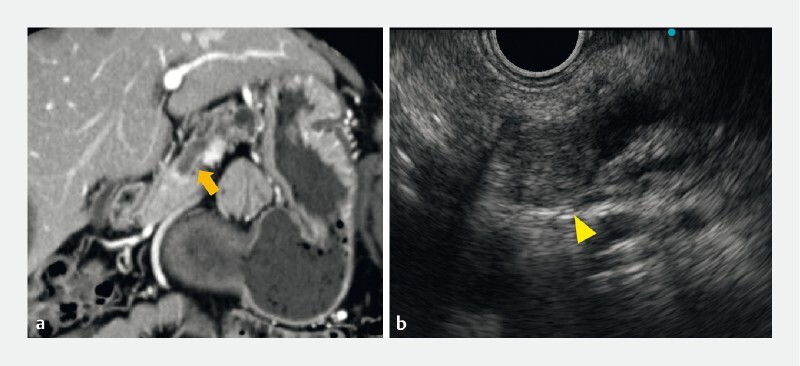
Computed tomography revealed an enhanced intraductal papillary tumor with main pancreatic duct dilation from the pancreatic body to the tail (arrow). **b** A hypoechoic papillary tumor in the main pancreatic duct was detected through endoscopic ultrasound (arrowhead).

**Fig. 3 a:**
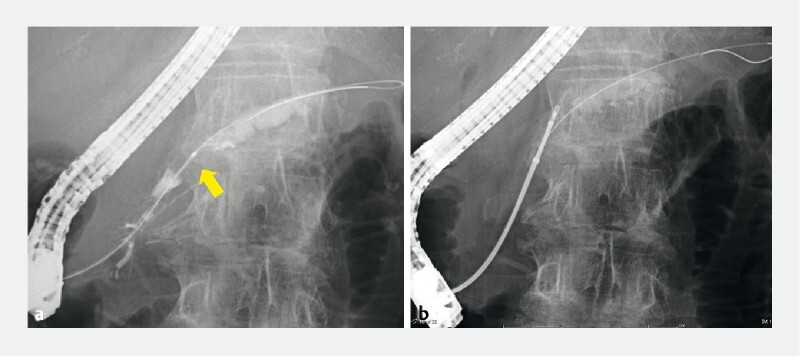
An intraductal pancreatic tumor was detected via pancreatography (arrow). **b** Forceps were advanced into the main pancreatic duct through the novel sheath device to obtain tumor tissues.

**Video 1** We successfully obtained considerable tissue samples of intraductal papillary mucinous carcinoma with pancreatic parenchyma invasion using the novel sheath device.


**Fig. 4 a:**
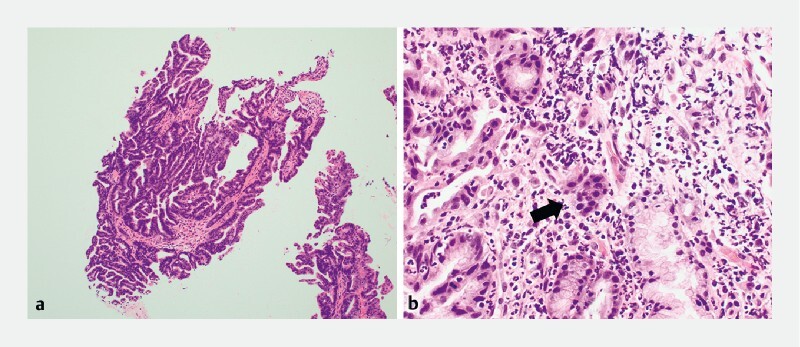
Histopathological examination showed atypical glands with irregular papillary and villoglandular structures lined with columnar epithelium. **b** Tumor cells spread into the stroma of the pancreas (arrow).

Endoscopy_UCTN_Code_TTT_1AR_2AD

## Notes

Endoscopy E-Videos

https://eref.thieme.de/e-videos
*E-Videos* is an open access online section of the journal *Endoscopy* , reporting on interesting cases and new techniques in gastroenterological endoscopy. All papers include a high-quality video and are published with a Creative Commons CC-BY license. Endoscopy E-Videos qualify for HINARI discounts and waivers and eligibility is automatically checked during the submission process. We grant 100% waivers to articles whose corresponding authors are based in Group A countries and 50% waivers to those who are based in Group B countries as classified by Research4Life (see: https://www.research4life.org/access/eligibility/ ). This section has its own submission website at https://mc.manuscriptcentral.com/e-videos
